# Smartphones vs Wearable Devices for Remotely Monitoring Physical Activity After Hospital Discharge: A Secondary Analysis of a Randomized Clinical Trial

**DOI:** 10.1001/jamanetworkopen.2019.20677

**Published:** 2020-02-07

**Authors:** Mitesh S. Patel, Daniel Polsky, Edward H. Kennedy, Dylan S. Small, Chalanda N. Evans, Charles A. L. Rareshide, Kevin G. Volpp

**Affiliations:** 1 Perelman School of Medicine, University of Pennsylvania, Philadelphia; 2 Wharton School, University of Pennsylvania, Philadelphia; 3 Penn Medicine Nudge Unit, University of Pennsylvania, Philadelphia; 4 Leonard Davis Institute for Health Economics, University of Pennsylvania, Philadelphia; 5 LDI Center for Health Incentives and Behavioral Economics, University of Pennsylvania, Philadelphia; 6 Department of Medicine, Crescenz Veterans Affairs Medical Center, Philadelphia, Pennsylvania; 7 Department of Health Policy and Management, Johns Hopkins University, Baltimore, Maryland; 8 Department of Statistics and Data Science, Carnegie Mellon University, Pittsburgh, Pennsylvania

## Abstract

This secondary analysis of a randomized clinical trial compares smartphones with wearable devices for remotely monitoring the duration of physical activity of patients after hospital discharge to home.

## Introduction

Nearly 80% of US adults own a smartphone,^1^ which accurately tracks physical activity.^2^ Wearable devices are growing in adoption and can track other biometrics.^2,3,4^ However, it is unknown whether smartphones or wearables are more sustainable for remotely monitoring patients over longer-term periods. The objective of this study was to compare the duration of remotely monitoring physical activity from smartphones vs wearables in the 6 months after hospital discharge.

## Methods

This secondary analysis of a randomized clinical trial was approved by the University of Pennsylvania institutional review board and followed the Consolidated Standards of Reporting Trials (CONSORT) reporting guideline. Data were obtained from an ongoing trial that enrolled patients from January 23, 2017, to January 7, 2019.^5^ Patients provided written informed consent to participate in the trial.

Adults admitted to medicine services at 2 hospitals in Philadelphia, Pennsylvania, were eligible if they could ambulate, had a smartphone compatible with the Withings HealthMate application, and planned to be discharged to home. Patients were randomly assigned to use the smartphone alone or with a wearable (Withings Steel) for 6 months (eFigure in the Supplement). Physical activity data from devices were obtained using Way to Health as described in a previous study.^6^ All patients received $50 to enroll and $50 to complete the trial. Patients in the smartphone group were given the wearable after trial completion. All patients selected communication preferences (text message, email, or telephone voice recording) and were sent a notification to synchronize their device if data had not been transmitted for 4 consecutive days.

For each patient, the duration of data transmission was estimated using the last day a step value was received and compared at 30, 90, and 180 days using Pearson χ^2^ tests. A Cox proportional hazard model was fit and adjusted for age, gender, race/ethnicity, insurance, education, marital status, annual household income, body mass index, and Charlson Comorbidity Index score; censoring took place on patient death. Because patients may not have synchronized data for all days, the proportion of days of data transmission was also compared using a χ^2^ test.

The intention-to-treat analysis was conducted in SAS software version 9.4 (SAS Institute) and used 2-sided hypothesis tests (level of significance, *P* < .05). Investigators and analysts were blinded to group assignment.

## Results

The sample comprised 500 patients (250 using smartphones and 250 using wearables) with a mean (SD) age of 46.6 (13.7) years; 320 (64%) were women, 219 (44%) white, 231 (46%) black, 141 (28%) enrolled in Medicare, and 128 (26%) enrolled in Medicaid (Table). Rates of patient death (smartphones, 5 patients; wearables, 7 patients) and overall dropout including death (smartphones, 7 patients; wearables, 12 patients) were similar.

**Table.** Sample Characteristics

Characteristic	No. (%)	
	Smartphone (n = 250)	Wearable Device (n = 250)
Age, y		
Mean (SD)	46.2 (13.6)	46.9 (13.9)
18-34	61 (24.4)	56 (22.4)
35-49	72 (28.8)	75 (30.0)
50-64	100 (40.0)	94 (37.6)
65-79	17 (6.8)	25 (10.0)
Women	155 (62.0)	165 (66.0)
Race/ethnicity		
Non-Hispanic		
White	102 (40.8)	117 (46.8)
Black	121 (48.4)	110 (44.0)
Hispanic	17 (6.8)	11 (4.4)
Other	10 (4.0)	12 (4.8)
Insurance		
Commercial	118 (47.2)	113 (45.2)
Medicare	71 (28.4)	70 (28.0)
Medicaid	61 (24.4)	67 (26.8)
Annual household income, $		
<50 000	84 (33.6)	84 (33.6)
50 000-100 000	41 (16.4)	40 (16.0)
>100 000	39 (15.6)	37 (14.8)
Patient declined to provide	86 (34.4)	89 (35.6)
Marital status		
Single	117 (46.8)	106 (42.4)
Married	92 (36.8)	99 (39.6)
Other	41 (16.4)	45 (18.0)
Education		
Less than high school graduate	14 (5.6)	25 (10.0)
High school graduate	150 (60.0)	146 (58.4)
College graduate	86 (34.4)	79 (31.6)
Body mass index, mean (SD)^a^	30.4 (8.4)	31.4 (9.6)
Charlson Comorbidity Index score, median (IQR)	3 (1-6)	3.5 (1-5)

Abbreviation: IQR, interquartile range.

^a^ Calculated as weight in kilograms divided by height in meters squared.

The proportion of patients transmitting data among the smartphone group was not different than among the wearable group after 30 days (86.7% vs 81.9%; difference, 4.9 percentage points; 95% CI, −1.5 to 11.3 percentage points; *P* = .13) but was significant at 90 days (77.6% vs 67.6%; difference, 9.9 percentage points; 95% CI, 2.1 to 17.8 percentage points; *P* = .01) and 180 days (61.2% vs 46.5%; difference, 14.7 percentage points; 95% CI, 6.0 to 23.5 percentage points; *P* = .001) (Figure). Patients in the smartphone group transmitted data for a significantly greater proportion of days during the 180-day period than patients in the wearable group (69.4% vs 58.9%; difference, 10.5 percentage points; 95% CI, 4.4 to 17.8 percentage points; *P* = .001).

**Figure.**
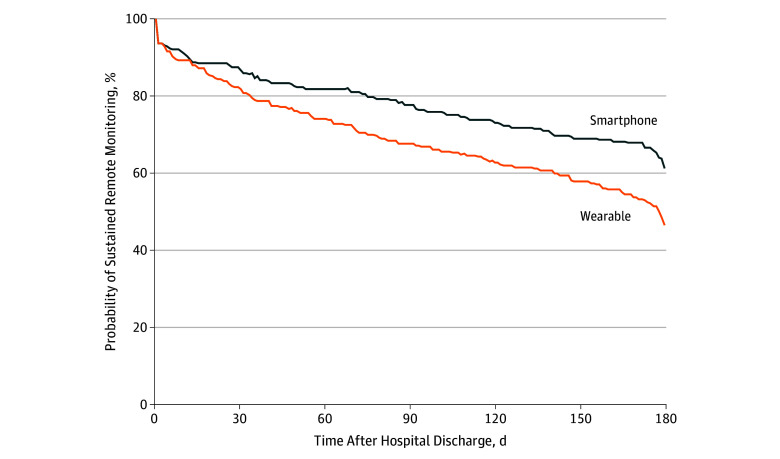
Duration of Sustained Remote Monitoring of Physical Activity Data After Hospital Discharge to Home The graph shows the percentage of patients who transmitted step count data to the study during the 180-day period. Patients in both groups received a reminder to synchronize their device if data had not been received for 4 consecutive days. Duration is defined as the last day a step value was received. Patients were censored on death (5 patients in the smartphone group and 7 in the wearable-device group). Patients who dropped out of the study because they were no longer interested were classified as no longer transmitting data but were not censored (2 patients in the smartphone group and 5 in the wearable-device group).

In the multivariate model, differences in duration remained significant, with lower discontinuation among the smartphone group (hazard ratio, 0.66; 95% CI, 0.50-0.86; *P* = .002). Being male was associated with less likelihood of discontinuation (hazard ratio, 0.71; 95% CI, 0.53-0.95; *P* = .02), and Medicare insurance was associated with greater likelihood of discontinuation (hazard ratio, 2.05; 95% CI, 1.41-2.96; *P* < .001).

## Discussion

Patients discharged from the hospital using smartphones transmitted data for a greater duration and proportion of time, with a 32% relative increase in patients completing the 180-day period compared with those using wearables. This study is limited to physical activity data from patients at 1 health system. Wearables track behaviors that smartphones do not (eg, sleep), and future research will evaluate the usefulness of these data.^5^ Because smartphones are ubiquitious,^1^ our findings indicate that these devices could be a scalable approach for remotely monitoring patient health behaviors.

## Notes

Supplement. **eFigure.** CONSORT Diagram
