# Supplementary material for: Smartphones vs Wearable Devices for Remotely Monitoring Physical Activity After Hospital Discharge: A Secondary Analysis of a Randomized Clinical Trial
Source: JAMA Netw Open. 2020 Feb 7;3(2):e1920677. doi: 10.1001/jamanetworkopen.2019.20677 (PMC12543400; doi:10.1001/jamanetworkopen.2019.20677)
Supplement: Supplement. — eFigure. CONSORT Diagram [file jamanetwopen-e1920677-s001.pdf]

Supplementary Online Content

Patel MS, Polsky D, Kennedy EH, et al. Smartphones vs wearable devices for remotely monitoring physical activity after hospital discharge: a secondary analysis of a randomized clinical trial. *JAMA Netw Open*. 2020;3(2):e1920677.
doi:10.1001/jamanetworkopen.2019.20677

eFigure. CONSORT Diagram

This supplementary material has been provided by the authors to give readers additional information about their work.

© 2020 Patel MS et al. *JAMA Network Open.*

eFigure. CONSORT Diagram

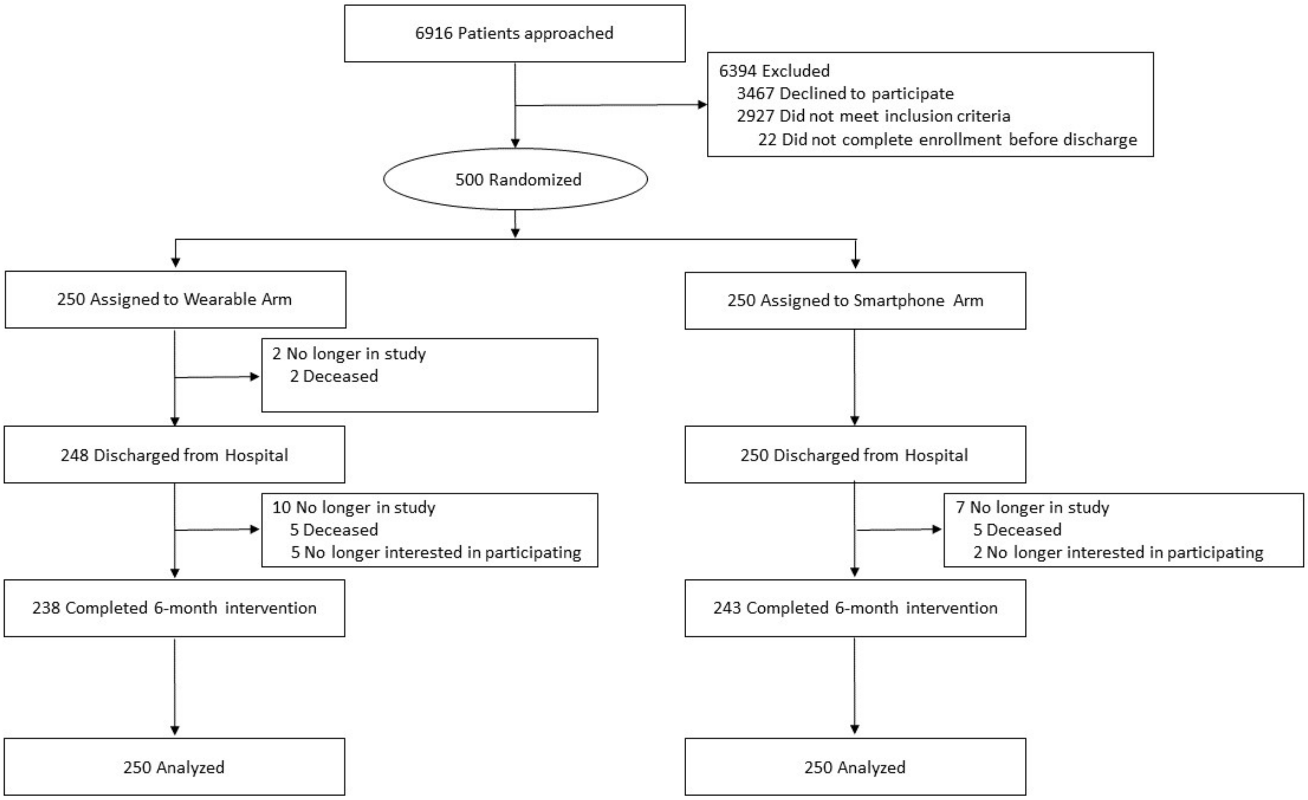

© 2020 Patel MS et al. *JAMA Network Open.*
